# Distribution and Genetic Characteristics of SXT/R391 Integrative Conjugative Elements in *Shewanella* spp. From China

**DOI:** 10.3389/fmicb.2018.00920

**Published:** 2018-05-11

**Authors:** Yujie Fang, Yonglu Wang, Zhenpeng Li, Zongdong Liu, Xinyue Li, Baowei Diao, Biao Kan, Duochun Wang

**Affiliations:** ^1^State Key Laboratory of Infectious Disease Prevention and Control, National Institute for Communicable Disease Control and Prevention, Chinese Center for Disease Control and Prevention, Beijing, China; ^2^Collaborative Innovation Center for Diagnosis and Treatment of Infectious Diseases, Hangzhou, China; ^3^Center for Human Pathogen Collection, Chinese Center for Disease Control and Prevention, Beijing, China; ^4^Ma’anshan Center for Disease Control and Prevention, Ma’anshan, China; ^5^Laizhou Center for Disease Control and Prevention, Laizhou, China

**Keywords:** distribution, genetic characteristics, SXT/R391, integrative conjugative elements, *Shewanella*, China

## Abstract

The genus *Shewanella* consists of facultatively anaerobic Gram-negative bacteria, which are regarded as potential agents of food contamination and opportunistic human pathogens. Information about the distribution and genetic characteristics of SXT/R391 integrative conjugative elements (ICEs) in *Shewanella* species is limited. Here, 91 *Shewanella* strains collected from diverse samples in China were studied for the presence of SXT/R391 ICEs. Three positive strains, classified as *Shewanella upenei*, were obtained from patients and water from a local mill. In light of their close clonal relationships and high sequence similarity, a representative ICE was selected and designated ICE*Sup*CHN110003. The BLASTn searches against GenBank showed that ICE*Vch*Ban5 was most closely related to ICE*Sup*CHN110003, with the coverage of 76% and identity of 99%. The phylogenetic tree of concatenated core genes demonstrated that ICE*Sup*CHN110003 formed a distinct branch outside the cluster comprising ICE*Val*A056-1, ICE*Pmi*CHN2410, and ICE*Pmi*Chn1. Comparison of the genetic structures revealed that ICE*Sup*CHN110003 encoded uncommon genes in hotspots, such as specific type III restriction-modification system, conferring adaptive functions to the host. Based on the low coverage in the sequence analysis, independent clade in the phylogenetic tree, and unique inserted fragments in hotspots, ICE*Sup*CHN110003 represented a novel SXT/R391 element, which widened the list of ICEs. Furthermore, the antibiotic resistance genes *floR*, *strA*, *strB*, and *sul2* in ICE*Sup*CHN110003 and resistance to multiple drugs of the positive isolates were detected. A cross-species transfer capability of the SXT/R391 ICEs was also discovered. In summary, it is necessary to reinforce continuous surveillance of SXT/R391 ICEs in the genus *Shewanella*.

## Introduction

In the last two decades, it has become known that horizontal gene transfer (HGT) plays a leading role in the evolutionary and environmental adaptation of bacteria (Hacker and Kaper, 2000; Juhas et al., 2009). The significance of HGT is ascribed to mobile genetic elements (MGEs) (Wozniak and Waldor, 2010). In essence, MGEs are discrete DNA segments involved in the transmission of various genes and constitute a crucial driving force in bacterial evolution. Previously described conjugative transposons, prophages, and integrative conjugative elements (ICEs) are members of the MGE family (Juhas et al., 2009). Among them, ICEs comprise a large number of self-transmissible elements, which can integrate into bacterial chromosomes, excise from their host chromosome, form a transient circular intermediate, transfer to another cell and reintegrate into the new host’s chromosome at the target site (Burrus et al., 2006; Wozniak and Waldor, 2010).

SXT/R391 elements belong to an ICE family, and to date, at least 89 ICEs from the SXT/R391 family have been identified^1^ (Bi et al., 2012). SXT/R391 ICEs have been found in different sources, including clinical, food, and environmental samples, and they share a conserved integrase that mediates the integration into the 5′ end of the *prfC* gene in the host chromosome (Burrus et al., 2006; Wozniak et al., 2009). SXT is an ∼100 kb ICE, which was originally detected in *Vibrio cholerae* O139 (Waldor et al., 1996), and R391 (89 kb) was identified in *Providencia rettgeri* in 1972 (Coetzee et al., 1972). SXT and R391 have been grouped in an ICE family containing 52 nearly identical core genes. Some of them are involved in the process of integration/excision, conjugative transfer, and regulation, while other core genes may encode functions that enhance ICE fitness to the environment. In addition to the core modules, five hotspots (HS1–5) and four variable regions (VRI–VRIV) have been reported, which contain inserted variable genes conferring resistance to antibiotics and heavy metals (Wozniak et al., 2009; Lei et al., 2016).

The genus *Shewanella* includes motile Gram-negative bacilli, which are widely distributed in marine habitats (Ronconi et al., 1999; Dikow, 2011). Currently, more than 60 species of *Shewanella* have been recognized^2^. They have been isolated from diverse samples and identified as potential agents of food contamination and opportunistic pathogens of humans. Among them, four species have been reported to cause human infections, i.e., *Shewanella putrefaciens*, *Shewanella algae*, *Shewanella haliotis*, and *Shewanella xiamenensis*. The majority of *Shewanella*-associated syndromes involve skin and soft-tissue infections (Pagani et al., 2003; Goyal et al., 2011; Rouzic et al., 2012), followed by blood borne illnesses (Dominguez et al., 1996) and infections of the biliary tree (Liu et al., 2013; Janda and Abbott, 2014).

The first complete SXT/R391 ICE reported in *Shewanella* species was a novel R391-like element, ICE*Spu*PO1, derived from *S. putrefaciens* W3-18-1, which was isolated from the Pacific Ocean 10 years ago (Pembroke and Piterina, 2006). SXT/R391 ICEs in the genus *Shewanella* have also been detected in *S. haliotis* in Portugal (Rodriguez-Blanco et al., 2012) and *S. fidelis* in Japan (Nonaka et al., 2012). ICE*Sha*Por1 of *S. haliotis*, available in GenBank, is only an amplified fragment of specific hotspot regions. The SXT/R391 element from the species *S. fidelis* has merely been documented by Nonaka et al. (2014), and the lack of public submission makes it impossible to compare. Recently, several novel SXT/R391 ICEs have been reported among *Vibrio* (Song et al., 2013; Wang et al., 2016) and *Proteus* (Lei et al., 2016; Li et al., 2016) species in China. However, little information is available on the distribution and genetic characteristics of SXT/R391 ICEs from *Shewanella* species in China. In this study, we screened 91 *Shewanella* strains, which were isolated between 2007 and 2016 in China, by targeting the *int*_SXT_ gene. We also investigated the sequence similarities, evolutionary relationships and genetic structures of novel SXT/R391 ICEs from *Shewanella* species. Further, antibiotic susceptibility of the positive isolates and the transfer capability of the SXT/R391 ICEs were determined.

## Materials and Methods

### Bacterial Isolation, Taxonomic Identification, and PFGE Characterization of *Shewanella* Strains

In this study, a total of 91 *Shewanella* isolates obtained from diverse sources were included. The collections of *Shewanella* isolates comprised of fecal specimens of diarrhea patients (*n* = 45), food samples (*n* = 39), and environments (*n* = 7) from four provinces (Anhui, Hainan, Liaoning, and Shandong) in China during the years 2007–2016. Pure bacterial cultures with pink–orange colored colonies were obtained on LB agar according to standard procedures described previously (Lei et al., 2016; Li et al., 2016). The taxonomy of *Shewanella* isolates was identified by the amplification and sequencing of 16S rRNA with universal primers 27F and 1492R (Lane, 1991). The *Shewanella* isolates were further characterized by pulsed field gel electrophoresis (PFGE) after the genomic DNA digestion of *Xba*I (Fermentas, United States) (Cooper et al., 2006). The results of PFGE were analyzed by Bionumerics software (Applied Maths, Belgium) to estimate the clonal relationships between different *Shewanella* isolates.

### PCR Screening, Genome Sequencing, and Assembly of SXT/R391-Harboring *Shewanella* Isolates

All *Shewanella* isolates were subjected to a PCR screen for SXT/R391 ICEs by targeting the *int*_SXT_ gene, which is regarded as a conserved integrase-coding gene among members of the ICE family. The PCR primers designed for the *int*_SXT_ gene were used as previously described (McGrath et al., 2006). Genomic DNA of SXT/R391-positive *Shewanella* isolates was extracted by the Wizard Genomic DNA Purification kit (Promega, United States) in line with the manufacturer’s protocols. The whole genomes were sequenced on the Illumina HiSeq 2000 platform, employing two paired-end libraries with average insert lengths of 500 and 2000 bp, respectively. The clean paired-end read data were assembled by means of SOAPdenovo v2.04 (Li et al., 2010). Genomic similarities of the SXT/R391-positive isolates were estimated by the average nucleotide identity (ANI) service^3^.

### Extraction, Annotation, and Submission of SXT/R391 ICEs

The SXT/R391 ICEs of the *Shewanella* isolates were identified and extracted by the alignment with the reference ICE sequence of SXT^MO10^ (AY055428.1). The complete ICEs of the isolates were assembled and obtained by PCR linkage of the gaps between separate scaffolds. Putative coding sequences of the SXT/R391 ICEs were predicted by Glimmer and annotated by the RAST (Rapid Annotation using Subsystem Technology) server (Aziz et al., 2008; Overbeek et al., 2014; Brettin et al., 2015). The integrated SXT/R391 ICE sequence and annotation of the representative *Shewanella* isolate were deposited in GenBank.

### Sequence Similarities and Phylogenetic Analysis of SXT/R391 Elements

The genetic and evolutionary relationships between the novel SXT/R391 of typical *Shewanella* isolate and the recognized SXT/R391 ICEs were determined by BLASTn search and phylogenetic analysis. The BLASTn tool was employed to obtain the similarities in nucleotide sequences of SXT/R391 elements and seek out the known ICEs with high homology. Based on blast results, 24 representative ICEs were selected with different sequences scores and distinct evolutionary origins to construct the phylogenetic tree of core genes (Supplementary Table S1). The concatenated sequences of core genes identified by OrthoMCL software were included and aligned in phylogenetic analysis. Phylogenetic tree was constructed by the maximum-likelihood method and evaluated with 1000 bootstrap replications.

### Comparative Analysis and Genetic Organization of the Novel SXT/R391 and Closely Related ICEs

The SXT/R391 sequence of the *Shewanella* isolate was further evaluated by comparison with classical and related SXT/R391 ICEs, i.e., SXT^MO10^ (*V. cholerae* MO10; AY055428.1), R391 (*P. rettgeri* R391; AY090559.1), ICE*Vch*Ban5 (*V. cholerae* Ban5; GQ463140.1), ICE*Spu*PO1 (*S. putrefaciens* W3-18-1; CP000503.1), and ICE*Sha*Por1 (*S. haliotis* AC6; HE577620.1). The sequence visualization of BLASTn analysis with four reference sequences listed above, except for ICE*Sha*Por1, which was assembled as PCR fragments of hotspot regions, was performed by the Artemis Comparison Tool (ACT) (Carver et al., 2008). Genetic structures of the novel SXT/R391 element from the *Shewanella* isolate and the five related ICEs were indicated for conserved and variable regions to distinguish unique genes among the SXT/R391 ICEs.

### Antibiotic Susceptibility Testing

The broth microdilution method was employed to determine the susceptibility of SXT/R391-harboring *Shewanella* isolates to 16 antibiotics, including amoxicillin/clavulanic acid, ampicillin, azithromycin, cefixime, cefoxitin, ceftriaxone, chloramphenicol, ciprofloxacin, doxycycline, gentamicin, imipenem, kanamycin, streptomycin, sulfamethoxazole, tetracycline, and trimethoprim-sulfamethoxazole. The standards of the Clinical and Laboratory Standards Institute (2015) were followed to carry out the antibiotic tests and identify the susceptibility of isolates.

### Conjugation Assays

The ability to transfer of the positive *Shewanella* isolates was estimated by the mating assays according to previous description (Mata et al., 2011). The streptomycin-resistant SXT/R391-positive *Shewanella* isolates were used as the donor isolates and the kanamycin-resistant *E. coli* SM10 served as the recipient isolate. Transconjugants were discerned from the selective media, which contained 100 μg/ml streptomycin and 100 μg/ml kanamycin. The transfer frequency of ICEs was determined by calculating the number of transconjugants per recipient cell. The transconjugants were further verified by the amplification of *int*_SXT_ gene and antibiotic resistance genes in the SXT/R391-harboring *Shewanella* isolates as previously described (Li et al., 2016).

## Results

### Distribution and Characterization of SXT/R391-Positive *Shewanella* Isolates

**FIGURE 1:**
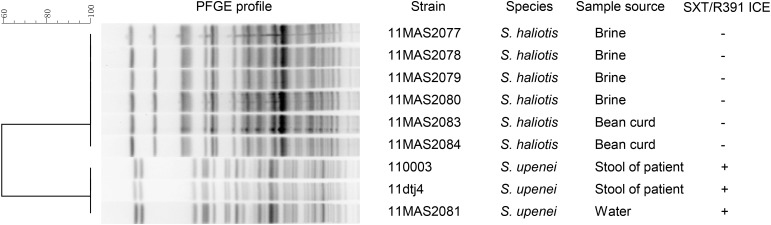
PFGE profile characterization of nine *Shewanella* strains isolated simultaneously from a local mill in Dangtu county, Anhui province, China. PFGE were performed with CHEF-DR III system under the following conditions: voltage gradient, 6 V/cm; run time, 20 h; initial switch time, 1 s; final switch time, 15 s.

The *Shewanella* strains in China belonged to seven species, including *S. algae*, *S. chilikensis*, *S. haliotis*, *S. indica*, *S. seohaensis*, *S. upenei*, and *S. xiamenensis*. SXT/R391-like ICEs were detected in 3 out of 91 *Shewanella* isolates, which belonged to the species *S. upenei* (Supplementary Figure S1 and Supplementary Table S2). Among the SXT/R391-harboring isolates, two were obtained from stool samples of diarrhea patients, and the third one was isolated from washing water. Interestingly, the three SXT/R391-positive isolates were derived from a local mill of soybean products in Dangtu county, Anhui province, China, on September 8, 2011. In the mill, another six *Shewanella* isolates were obtained from the brine and bean curd; however, these isolates were negative for SXT/R391 elements and were confirmed as strains of *S. haliotis* (Supplementary Figure S1). Based on the PFGE analysis, which revealed the clonal relationships of the nine isolates from Dangtu county, the three SXT/R391-harboring isolates of *S. upenei* had similar profiles, and the six SXT/R391-negative isolates of *S. haliotis* shared identical patterns, traced to the same origin, respectively (**Figure 1**).

### Sequence Analysis and ICE Features of SXT/R391 in *Shewanella* Isolates

The assembled genomes of the three SXT/R391-positive isolates showed high similarity values (99.9%), as estimated by ANI. Analysis of the three complete SXT/R391 elements showed that the ICE sequences were almost identical, with only several different bases in variable regions. Given that the three ICE-harboring isolates shared identical PFGE profiles, high genomic similarities, and nuances of SXT/R391 elements, strain 110003 was selected as the representative SXT/R391-positive *Shewanella* isolate. The corresponding SXT/R391 element was designated ICE*Sup*CHN110003 following the nomenclature for the ICE family. The sequence of ICE*Sup*CHN110003 was 91,669 bp in length, with 46.3% GC content, included 82 coding genes (Supplementary Table S3) and was submitted in GenBank under accession number MG014393.

### BLAST Results for ICE*Sup*CHN110003 and ICEs Available in GenBank

The nucleotide sequence of ICE*Sup*CHN110003 was aligned with those of the ICEs available in the GenBank database to evaluate its homology with the recognized ICEs. Comparison between ICE*Sup*CHN110003 and the reported ICEs showed a query coverage from 40 to 76%, the identity from 96 to 99%, the total score from 64,604 to 1.89E+05 and the max score from 15,189 to 46,405. The most closely related ICE was ICE*Vch*Ban5 carried by *V. cholerae* strain Ban5, with the query coverage of 76% and identity of 99%. However, the novel SXT/R391 element from the *Shewanella* isolate exhibited relatively low homology to classical ICEs, i.e., SXT^MO10^ and R391, with 70 and 54% query coverages as well as 97 and 98% identities, respectively. Comparison between ICE*Sup*CHN110003 and ICE*Spu*PO1 from *S. putrefaciens* showed that the query coverage and identity were 57 and 97%, respectively.

### Taxonomic Position of ICE*Sup*CHN110003

**FIGURE 2:**
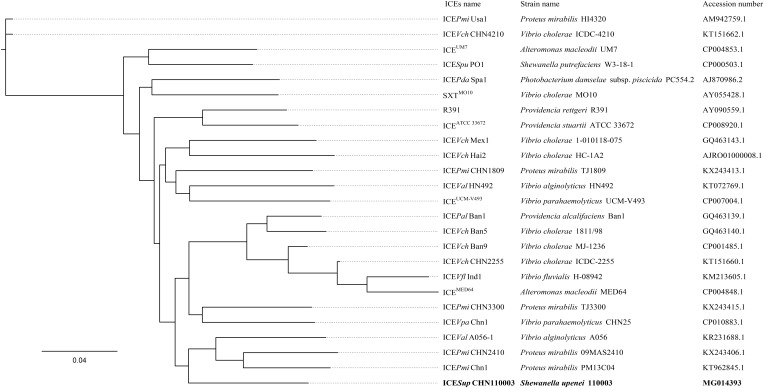
Phylogenetic analysis based on the concatenated core genes between ICE*Sup*CHN110003 and 24 representative ICEs. The phylogenetic tree was constructed by maximum-likelihood methods. Bootstrap values were evaluated with 1000 replications. Bold font indicated the ICE*Sup*CHN110003.

The phylogenetic tree of concatenated core genes demonstrated that ICE*Sup*CHN110003 formed an independent branch outside the cluster comprising of ICE*Val*A056-1 (Guangdong, China, 2003), ICE*Pmi*CHN2410 (Anhui, China, 2009), and ICE*Pmi*Chn1 (Hubei, China, 2013) (**Figure 2** and Supplementary Table S1). The strains in the cluster involving *Vibrio alginolyticus* and *Proteus mirabilis* species were isolated from various sources including shrimp, chicken, and stool specimen of patient. The phylogenetic analysis indicated that the ICE*Sup*CHN110003 as a novel SXT/R391 element was evolutionarily related to the three ICEs discovered in China.

### Overall Comparison Between ICE*Sup*CHN110003 and Representative Related ICEs

**FIGURE 3:**
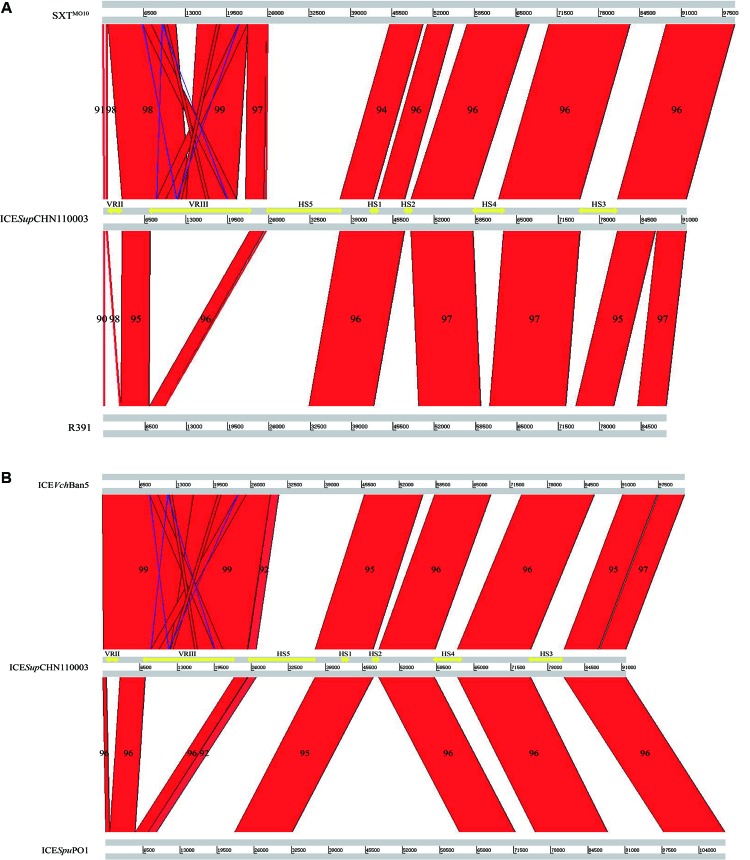
General comparisons presented by ACT software of ICE*Sup*CHN110003 with: **(A)** SXT^MO10^ and R391; **(B)** ICE*Vch*Ban5 and ICE*Spu*PO1. Red areas indicated homologous regions. Blue areas indicated inverse regions. Yellow arrows indicated hotspots and variable regions in ICE*Sup*CHN110003. Numbers in areas indicated the similarity (%) of the compared regions.

By and large, collinear relationships among the five ICEs were detected in conserved regions and variable region III (**Figure 3**). ICE*Sup*CHN110003 with a complete core backbone exhibited 94–97% similarity with the reference ICEs in conserved regions. Higher similarities among the ICE sequences (98–99%) could be found in variable region III, which existed in ICE*Sup*CHN110003, SXT^MO10^, and ICE*Vch*Ban5. Inverted areas were also observed in this region, corresponding to several genes of mobile elements. Unmatched areas were concentrated in specific inserted variable regions with low homology, making ICE*Sup*CHN110003 a novel SXT/R391 element that could be distinguished from the recognized ICEs.

### Genetic Structure of Hotspots and Variable Regions in SXT/R391 ICEs

**FIGURE 4:**
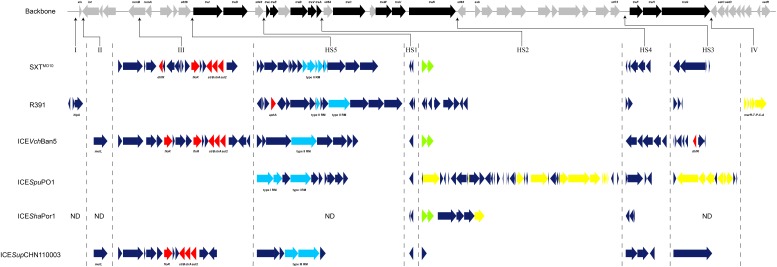
Genetic structure of ICE*Sup*CHN110003 and five representative ICEs: SXT^MO10^, R391, ICE*Vch*Ban5, ICE*Spu*PO1, and ICE*Sha*Por1. The upper line demonstrated the backbone of 52 core genes in SXT/R391 ICEs. Black arrows indicated the coding genes involved in conjugative transfer process. Gray arrows indicated other genes in conserved regions. Under the backbone, the inserted genes of six SXT/R391 ICEs in five hotspots (HS1–5) and four variable regions (I–IV) were shown. Red arrows indicated antibiotic resistance genes. Light blue arrows indicated restriction-modification system. Green arrows indicated toxin–antitoxin system. Yellow arrows indicated heavy metals resistance genes. Dark blue arrows indicated other genes in hotspots and variable regions. Several distinct genes in these regions were marked. ND, not determined.

Analysis of genetic organization was performed among the aforementioned SXT/R391 sequences, with the addition of ICE*Sha*Por1 from *S. haliotis*, which was amplified in specific inserted loci (**Figure 4**). The genetic structure of ICE*Sup*CHN110003 in inserted regions indicated that the novel SXT/R391 element from the *Shewanella* isolate comprised five hotspots (HS1–5) and two variable regions (II and III).

The five intergenic hotspot regions were identified by locating the boundaries, as described previously (Wozniak et al., 2009). In HS1, a conserved hypothetical gene of 588 bp in length was identified in ICE*Sup*CHN110003, which shared 94.7–96.6% similarity with the reference ICEs, with the exception of SXT^MO10^, consistent with the graphical alignment generated by the ACT software (**Figure 3**). In HS2, mosA/T toxin–antitoxin systems, which support ICE maintenance, or heavy metal efflux gene clusters, which protect against heavy metal toxicity, were identified in four reference ICEs apart from R391. Whereas, ICE*Sup*CHN110003 encoded a distinct gene exhibiting high homology to ICE*Vsc*Spa2 with only 2-bp difference, indicating their close evolutionary origin. This gene was found to share 49% similarity with the ProP osmoprotectant transporter gene from *Francisella tularensis* subsp. *holarctica* (Rodriguez-Blanco et al., 2012). In HS3, a large DNA fragment of approximately 5.7 kb, only discovered in ICE*Pmi*Chn2, was inserted in this region and annotated as a serine protease-like protein by RAST (Supplementary Table S3). Gene clusters encoding endonuclease, helicase, methyl-accepting chemotaxis protein, restriction-modification (RM) system and the *mrr* restriction system were identified in HS4 and HS5. Among the reference ICEs, a type I RM system was detected in ICE*Spu*PO1, and a type II RM system was discovered in SXT^MO10^, R391, and ICE*Vch*Ban5. On the other hand, the novel SXT/R391 ICE*Sup*CHN110003 encoded a type III RM system, which was differentiated from those of closely related ICEs. The type III RM system, comprising the R (restriction) subunit and the M (modification) subunit and conferring resistance to phage infection, was delineated in ICE*Vsp*Por3 and ICE*Val*Spa1 among the recognized ICEs (Balado et al., 2013). However, only few ICE*Sup*CHN110003 areas were aligned with those, indicating a distant relationship and diversity among the type III RM systems in SXT/R391 elements. The BLASTn results for the type III RM system of ICE*Sup*CHN110003 showed its higher homology with several complete genome and plasmid sequences, except for the M subunit, which had a nucleotide coverage of only approximately 50%. Because of the low similarities in type III RM systems among SXT/R391 elements and the half coverage of the M subunit in public databases, the RM system found in ICE*Sup*CHN110003 served as a distinct type III RM system in ICEs.

Two inserted variable regions (II and III) were identified in ICE*Sup*CHN110003, conferring adaptive functions and multidrug resistance to the isolate. The high level of homology (99%) in these regions between ICE*Sup*CHN110003 and ICE*Vch*Ban5, as shown by ACT, made ICE*Vch*Ban5 the most closely related ICE. Variable region II, located between the *xis* and *int* genes, included a DNA mismatch repair gene, *mutL*, which is involved in the process of DNA replication, recombination, and repair. Variable region III, disrupting the *rumB* gene, was detected in SXT^MO10^, ICE*Vch*Ban5, and ICE*Sup*CHN110003. A putative transposon cassette harboring multidrug resistance genes was present in this region, which tends to accumulate and disseminate antibiotic resistance, as previously described (Wozniak et al., 2009). In ICE*Sup*CHN110003, variable region III comprised four antibiotic resistance genes, including *floR*, *strA*, *strB*, and *sul2*, which mediate resistance to chloramphenicol, streptomycin, and sulfamethoxazole, respectively. By comparison, ICE*Vch*Ban5 possessed an extra copy of *floR*, and SXT^MO10^ carried the *dhfR* gene conferring resistance to trimethoprim. In general, the acquisition and recombination of genes, frequently occurring in hotspots and variable regions of ICEs, lead to the emergence of novel SXT/R391 elements such as ICE*Sup*CHN110003, stably maintained in a rapidly changing environment.

### Antibiotic Resistance of SXT/R391-Harboring *Shewanella* Isolates

The three SXT/R391-positive *Shewanella* isolates, 110003, 11dtj4, and 11MAS2081, were subjected to antibiotic susceptibility tests and found to share similar multidrug resistance profiles. All isolates were resistant to 9 out of 16 drugs, including amoxicillin/clavulanic acid, cefixime, cefoxitin, chloramphenicol, imipenem, streptomycin, sulfamethoxazole, tetracycline, and trimethoprim-sulfamethoxazole, except for intermediate resistance of isolate 110003 to tetracycline. The phenotype of multidrug resistance to chloramphenicol, streptomycin, sulfamethoxazole, and trimethoprim-sulfamethoxazole could be attributed to the presence of the SXT/R391 element, which carried the antibiotic resistance genes *floR*, *strA*, *strB*, and *sul2*.

### Transfer Ability of SXT/R391 Elements in *Shewanella* Isolates

A conjugation assay was employed to investigate the transfer ability of the three SXT/R391-harboring *Shewanella* isolates. The mobility of ICEs was detected, with a transfer frequency ranging from 9.5 × 10^-8^ (11dtj4) to 5 × 10^-7^ (110003) per recipient cell, indicating the possibility of gene dissemination via horizontal transfer of SXT/R391 ICEs. The transconjugants were further confirmed by PCR-based amplification targeting the *int*_SXT_ gene and antibiotic resistance genes (*floR*, *strA*, *strB*, and *sul2*) harbored by the SXT/R391-positive *Shewanella* isolates.

## Discussion

Until now, SXT/R391 ICEs have been investigated in the genera *Vibrio* (Wang et al., 2016), *Providencia* (Coetzee et al., 1972), *Photobacterium* (Nonaka et al., 2012), *Proteus* (Lei et al., 2016; Li et al., 2016), *Alteromonas* (Badhai and Das, 2016), *Enterovibrio* (Taviani et al., 2009; Song et al., 2013; Luo et al., 2016), and *Shewanella*. *S. putrefaciens*, *S. haliotis*, and *S. fidelis* in the genus *Shewanella*, which were obtained from the Pacific Ocean, Portugal, and Japan, respectively, were reported to harbor SXT/R391 elements (Pembroke and Piterina, 2006; Rodriguez-Blanco et al., 2012; Nonaka et al., 2014). In this study, we discerned seven *Shewanella* species, including *S. algae*, *S. chilikensis*, *S. haliotis*, *S. indica*, *S. seohaensis*, *S. upenei*, and *S. xiamenensis*, among 91 strains isolated from four provinces in China, whereas only the species *S. upenei* from Anhui province was found to carry SXT/R391 elements. This was the first time when SXT/R391 elements were detected in the genus *Shewanella* in China, and a novel multidrug-resistant ICE, ICE*Sup*CHN110003, was characterized in *S. upenei*. Three SXT/R391-positive strains of *S. upenei*, with similar PFGE profiles, were simultaneously isolated from fecal samples of diarrhea patients and from washing water. The existence of SXT/R391-harboring *Shewanella* isolates among patients and in an aquatic environment from an inland city raises risks of contaminating water sources and causing diseases. Six isolates of *S. haliotis*, which was reported to have the potential to acquire ICEs, were also obtained from commercial food at the same place and time. The SXT/R391 ICE*Sup*CHN110003 present in positive isolates as a self-transmissible element, was proven to have the ability to transfer via conjugation. It is likely that the ICEs harbored by *S. upenei* could transmit to *S. haliotis* isolates in food and to other aquatic bacteria in water and confer multidrug resistance to the hosts. As a consequence, it is urgent to reinforce continuous and widespread surveillance for ICEs in the genus *Shewanella*. Meanwhile, more attention should be paid to preventing the occurrence and dispersion of SXT/R391-harboring isolates.

The sequence relationships between ICE*Sup*CHN110003 and known ICEs available in public databases were evaluated by BLASTn, and the results showed that the nucleotide query coverage was less than 76% and the sequence similarity ranged from 96 to 99%. However, the graphical alignment generated by the ACT software demonstrated high levels of similarity with typical ICEs in conserved backbone genes, with 94–97% homology, and in variable region III, encoding multidrug resistance genes, with 98–99% similarity. The low coverage values were attributed to the unique genes positioned in hotspot regions of ICE*Sup*CHN110003, which were rarely discovered among recognized ICEs. Based on a comprehensive consideration of multiple indicators, the novel ICE shared a higher homology with ICE*Vch*Ban5 (Wozniak et al., 2009) from the genus *Vibrio* than with the previously reported ICEs from *Shewanella* species, indicating a low correlation within the genus.

The phylogenetic tree based on core genes demonstrated that ICE*Sup*CHN110003 formed a distinct clade separated from other representative ICEs. This evolutionary analysis revealed that ICE*Sup*CHN110003 was a novel ICE, adding to the members of the SXT/R391 family. The closely related ICEs were harbored by the species *V. alginolyticus* and *P. mirabilis*, indicating those as possible ancestors, rather than the SXT/R391 elements previously described in *Shewanella* species (Nonaka et al., 2014). Phylogenetic neighbors of ICEs were isolated from different provinces in China during a long time span, from 2003 to 2013. The discovery of ICE*Sup*CHN110003, emerged in 2011, reveals the evolution and dispersion of SXT/R391 ICEs in China. Notably, the related ICE*Pmi*CHN2410 (Li et al., 2016) was identified in *P. mirabilis* from the same province, Anhui, as the novel ICE. The close geographical distribution suggests an extensive local transfer of ICEs among diverse bacterial species. Therefore, a wide-ranging monitoring of ICEs is necessary to prevent the spread of related mobile elements in China.

Comparative genetic structure analysis was employed to elucidate the gene organization in hotspots and variable regions of the novel SXT/R391. The unique genes detected in five hotspots of ICE*Sup*CHN110003 had few counterparts in the NCBI database. For instance, the novel SXT/R391 harbored uncommon coding genes in the HS2 and HS3 regions, exclusively corresponding to those in ICE*Vsc*Spa2 and ICE*Pmi*Chn2, respectively. The distinct type III RM system found in ICE*Sup*CHN110003 showed low homology with those in recognized ICEs, whereas only partial fragments were discovered in genome and plasmid sequences. A novel functional gene cluster detected in ICE*Sup*CHN110003 endowed the hosts with adaptive abilities to the changing environment. Specific genes found in SXT/R391 elements from diverse origins indicate that the acquisition and recombination of foreign genes are common among ICEs, as previously described (Wozniak et al., 2009). Although unique accessory genes were detected in ICEs, genomes, and plasmids, none of the SXT/R391 elements were completely identical to ICE*Sup*CHN110003, owing to their mosaicism. Comparative analysis provided further evidence supporting characterization of ICE*Sup*CHN110003 as a novel SXT/R391 element distinguished from other ICEs.

An inserted fragment in variable region III contributed to the high level of homology between ICE*Sup*CHN110003 and ICE*Vch*Ban5, which was identified as the most closely related ICE. A multidrug resistance cassette was discovered in this region of the novel SXT/R391, conferring multiple resistance to antibiotics. The SXT/R391-positive *Shewanella* isolates exhibited a wide range of phenotypic resistance to more than half of the tested drugs. The antibiotic resistance determinants, including *floR*, *strA*, *strB*, and *sul2*, detected in ICE*Sup*CHN110003, are considered to confer resistance to chloramphenicol, streptomycin, and sulfamethoxazole. In addition, drug resistance genes located on the chromosome or a plasmid may endow the hosts with resistance to the remaining antibiotics. In addition, ICEs served as carriers for multidrug resistance clusters and transposase genes frequently occurred in variable region III. These transposable genetic elements in ICE*Sup*CHN110003 would promote HGT of antibiotic resistance determinants in clinical and aquatic environments.

## Conclusion

In conclusion, our study describes, for the first time, SXT/R391 elements detected in *Shewanella* species in China, which were isolated from the clinic, environment, and food. Three *Shewanella* isolates were found to harbor SXT/R391 elements, and ICE*Sup*CHN110003 was designated a representative ICE among the positive isolates. Given its low alignment coverage with recognized ICEs, independent clade in a phylogenetic tree of conserved core genes and uncommon inserted fragments in hotspots regions, ICE*Sup*CHN110003 represents a novel SXT/R391, thus enhancing the knowledge of ICEs. Resistance to multiple antibiotics and a cross-species transfer ability were discovered in these SXT/R391-positive *Shewanella* isolates from patients and water, posing a deep threat to public health and natural environments. Hence, it is imperative to increase our awareness of the emergence of ICEs in *Shewanella* species and take actions to prevent dissemination of SXT/R391 ICEs.

## Ethics Statement

This study was carried out in accordance with the recommendations of the Ethics Committee of National Institute for Communicable Disease Control and Prevention, Chinese Center for Disease Control and Prevention with written informed consent from all subjects. All subjects gave written informed consent in accordance with the Declaration of Helsinki. The protocol was approved by the Ethics Committee of National Institute for Communicable Disease Control and Prevention, Chinese Center for Disease Control and Prevention.

## Author Contributions

DW designed the work. YF, ZhL, BD, and XL performed the experiments. YW and ZoL collected the sample and isolated the strain. YF, ZhL, DW, and BK analyzed the data. YF and DW wrote the paper.

## Conflict of Interest Statement

The authors declare that the research was conducted in the absence of any commercial or financial relationships that could be construed as a potential conflict of interest.

## Footnotes

1 http://db-mml.sjtu.edu.cn/ICEberg/

2 http://www.bacterio.net/shewanella.html

3 http://www.ezbiocloud.net/tools/ani

## References

[B1] Aziz R. K. Bartels D. Best A. A. Dejongh M. Disz T. Edwards R. A. (2008). The RAST server: rapid annotations using subsystems technology. 9:75. 10.1186/1471-2164-9-75 18261238 PMC2265698

[B2] Badhai J. Das S. K. (2016). Characterization of three novel SXT/R391 integrating conjugative elements ICE*Mfu*Ind1a and ICE*Mfu*Ind1b, and ICE*Mpr*Chn1 identified in the genomes of *Marinomonas fungiae* JCM 18476T and *Marinomonas profundimaris* strain D104. 7:1896. 10.3389/fmicb.2016.01896 27933056 PMC5122569

[B3] Balado M. Lemos M. L. Osorio C. R. (2013). Integrating conjugative elements of the SXT/R391 family from fish-isolated *Vibrios* encode restriction-modification systems that confer resistance to bacteriophages. 83 457–467. 10.1111/1574-6941.12007 22974320

[B4] Bi D. Xu Z. Harrison E. M. Tai C. Wei Y. He X. (2012). ICEberg: a web-based resource for integrative and conjugative elements found in Bacteria. 40 D621–D626. 10.1093/nar/gkr846 22009673 PMC3244999

[B5] Brettin T. Davis J. J. Disz T. Edwards R. A. Gerdes S. Olsen G. J. (2015). RASTtk: a modular and extensible implementation of the RAST algorithm for building custom annotation pipelines and annotating batches of genomes. 5:8365. 10.1038/srep08365 25666585 PMC4322359

[B6] Burrus V. Marrero J. Waldor M. K. (2006). The current ICE age: biology and evolution of SXT-related integrating conjugative elements. 55 173–183. 10.1016/j.plasmid.2006.01.001 16530834

[B7] Carver T. Berriman M. Tivey A. Patel C. Bohme U. Barrell B. G. (2008). Artemis and ACT: viewing, annotating and comparing sequences stored in a relational database. 24 2672–2676. 10.1093/bioinformatics/btn529 18845581 PMC2606163

[B8] Clinical and Laboratory Standards Institute (2015). *Performance Standards for Antimicrobial Susceptibility Testing: Twenty-Fifth Informational Supplement. CLSI document M100-S25*. Wayne, PA: CLSI.

[B9] Coetzee J. N. Datta N. Hedges R. W. (1972). R factors from *Proteus rettgeri*. 72 543–552. 10.1099/00221287-72-3-543 4564689

[B10] Cooper K. L. Luey C. K. Bird M. Terajima J. Nair G. B. Kam K. M. (2006). Development and validation of a PulseNet standardized pulsed-field gel electrophoresis protocol for subtyping of *Vibrio cholerae*. 3 51–58. 10.1089/fpd.2006.3.51 16602979

[B11] Dikow R. B. (2011). Genome-level homology and phylogeny of *Shewanella* (Gammaproteobacteria: lteromonadales: Shewanellaceae). 12:237. 10.1186/1471-2164-12-237 21569439 PMC3107185

[B12] Dominguez H. Vogel B. F. Gram L. Hoffmann S. Schaebel S. (1996). *Shewanella alga* bacteremia in two patients with lower leg ulcers. 22 1036–1039. 10.1093/clinids/22.6.1036 8783706

[B13] Goyal R. Kaur N. Thakur R. (2011). Human soft tissue infection by the emerging pathogen *Shewanella algae*. 5 310–312. 10.3855/jidc.1436 21537075

[B14] Hacker J. Kaper J. B. (2000). Pathogenicity islands and the evolution of microbes. 54 641–679. 10.1146/annurev.micro.54.1.641 11018140

[B15] Janda J. M. Abbott S. L. (2014). The genus *Shewanella*: from the briny depths below to human pathogen. 40 293–312. 10.3109/1040841X.2012.726209 23043419

[B16] Juhas M. Van Der Meer J. R. Gaillard M. Harding R. M. Hood D. W. Crook D. W. (2009). Genomic islands: tools of bacterial horizontal gene transfer and evolution. 33 376–393. 10.1111/j.1574-6976.2008.00136.x 19178566 PMC2704930

[B17] Lane D. J. (1991). “16S/23S rRNA sequencing,” in , eds Stackebrandt E. Goodfellow M. (New York, NY: Wiley), 115–175.

[B18] Lei C. W. Zhang A. Y. Wang H. N. Liu B. H. Yang L. Q. Yang Y. Q. (2016). Characterization of SXT/R391 integrative and conjugative elements in *Proteus mirabilis* isolates from food-producing animals in China. 60 1935–1938. 10.1128/AAC.02852-15 26824957 PMC4775944

[B19] Li R. Zhu H. Ruan J. Qian W. Fang X. Shi Z. (2010). De novo assembly of human genomes with massively parallel short read sequencing. 20 265–272. 10.1101/gr.097261.109 20019144 PMC2813482

[B20] Li X. Du Y. Du P. Dai H. Fang Y. Li Z. (2016). SXT/R391 integrative and conjugative elements in Proteus species reveal abundant genetic diversity and multidrug resistance. 6:37372. 10.1038/srep37372 27892525 PMC5124997

[B21] Liu P. Y. Lin C. F. Tung K. C. Shyu C. L. Wu M. J. Liu J. W. (2013). Clinical and microbiological features of *Shewanella* bacteremia in patients with hepatobiliary disease. 52 431–438. 10.2169/internalmedicine.52.8152 23411697

[B22] Luo P. He X. Wang Y. Liu Q. Hu C. (2016). Comparative genomic analysis of six new-found integrative conjugative elements (ICEs) in *Vibrio alginolyticus*. 16:79. 10.1186/s12866-016-0692-9 27145747 PMC4857294

[B23] Mata C. Navarro F. Miro E. Walsh T. R. Mirelis B. Toleman M. (2011). Prevalence of SXT/R391-like integrative and conjugative elements carrying blaCMY-2 in *Proteus mirabilis*. 66 2266–2270. 10.1093/jac/dkr286 21752830

[B24] McGrath B. M. O’halloran J. A. Piterina A. V. Pembroke J. T. (2006). Molecular tools to detect the IncJ elements: a family of integrating, antibiotic resistant mobile genetic elements. 66 32–42. 10.1016/j.mimet.2005.10.004 16316703

[B25] Nonaka L. Maruyama F. Miyamoto M. Miyakoshi M. Kurokawa K. Masuda M. (2012). Novel conjugative transferable multiple drug resistance plasmid pAQU1 from *Photobacterium damselae* subsp. *damselae* isolated from marine aquaculture environment. 27 263–272. 10.1264/jsme2.ME11338 22446310 PMC4036041

[B26] Nonaka L. Maruyama F. Onishi Y. Kobayashi T. Ogura Y. Hayashi T. (2014). Various pAQU plasmids possibly contribute to disseminate tetracycline resistance gene tet(M) among marine bacterial community. 5:152. 10.3389/fmicb.2014.00152 24860553 PMC4026752

[B27] Overbeek R. Olson R. Pusch G. D. Olsen G. J. Davis J. J. Disz T. (2014). The SEED and the rapid annotation of microbial genomes using subsystems technology (RAST). 42 D206–D214. 10.1093/nar/gkt1226 24293654 PMC3965101

[B28] Pagani L. Lang A. Vedovelli C. Moling O. Rimenti G. Pristera R. (2003). Soft tissue infection and bacteremia caused by *Shewanella putrefaciens*. 41 2240–2241. 10.1128/JCM.41.5.2240-2241.2003 PMC154735 12734291

[B29] Pembroke J. T. Piterina A. V. (2006). A novel ICE in the genome of *Shewanella putrefaciens* W3-18-1: comparison with the SXT/R391 ICE-like elements. 264 80–88. 10.1111/j.1574-6968.2006.00452.x 17020552

[B30] Rodriguez-Blanco A. Lemos M. L. Osorio C. R. (2012). Integrating conjugative elements as vectors of antibiotic, mercury, and quaternary ammonium compound resistance in marine aquaculture environments. 56 2619–2626. 10.1128/AAC.05997-11 22314526 PMC3346659

[B31] Ronconi M. Merino L. Usandizaga G. B. Camargo M. C. Irigoyen B. I. Presti S. (1999). Non fermentative gram negative bacilli isolated in a hospital laboratory. 17 269–273. 10439535

[B32] Rouzic N. Hery-Arnaud G. Jaffuel S. Garo B. Payan C. Garre M. (2012). Soft tissue infection associated with bacteremia caused by *Shewanella putrefaciens*. 60 e27–e29. 10.1016/j.patbio.2011.04.005 21616609

[B33] Song Y. Yu P. Li B. Pan Y. Zhang X. Cong J. (2013). The mosaic accessory gene structures of the SXT/R391-like integrative and conjugative elements derived from *Vibrio* spp. isolated from aquatic products and environment in the Yangtze River Estuary, China. 13:214. 10.1186/1471-2180-13-214 24074349 PMC3850215

[B34] Taviani E. Grim C. J. Chun J. Huq A. Colwell R. R. (2009). Genomic analysis of a novel integrative conjugative element in *Vibrio cholerae*. 583 3630–3636. 10.1016/j.febslet.2009.10.041 19850044

[B35] Waldor M. K. Tschape H. Mekalanos J. J. (1996). A new type of conjugative transposon encodes resistance to sulfamethoxazole, trimethoprim, and streptomycin in *Vibrio cholerae* O139. 178 4157–4165. 10.1128/jb.178.14.4157-4165.1996 8763944 PMC178173

[B36] Wang R. Yu D. Yue J. Kan B. (2016). Variations in SXT elements in epidemic *Vibrio cholerae* O1 El Tor strains in China. 6:22733. 10.1038/srep22733 26956038 PMC4783696

[B37] Wozniak R. A. Fouts D. E. Spagnoletti M. Colombo M. M. Ceccarelli D. Garriss G. (2009). Comparative ICE genomics: insights into the evolution of the SXT/R391 family of ICEs. 5:e1000786. 10.1371/journal.pgen.1000786 20041216 PMC2791158

[B38] Wozniak R. A. Waldor M. K. (2010). Integrative and conjugative elements: mosaic mobile genetic elements enabling dynamic lateral gene flow. 8 552–563. 10.1038/nrmicro2382 20601965

