# Supplementary material for: Distribution and Genetic Characteristics of SXT/R391 Integrative Conjugative Elements in Shewanella spp. From China
Source: Front Microbiol. 2018 May 11;9:920. doi: 10.3389/fmicb.2018.00920 (PMC5958206; doi:10.3389/fmicb.2018.00920)
Supplement: Supplementary file 1 [file Table_1.doc]

**Supplementary Materials**

**Distribution and Genetic Characteristics of SXT/R391 Integrative Conjugative Elements in *Shewanella* spp. from China**

Yujie Fang^1,2,3^, Yonglu Wang^4^, Zhenpeng Li^1,2^, Zongdong Liu^5^, Xinyue Li^1,2^, Baowei Diao^1,2^, Biao Kan^1,2^, Duochun Wang^1,2,3^*

^1^State Key Laboratory for Infectious Disease Prevention and Control, National Institute for Communicable Disease Control and Prevention, Chinese Center for Disease Control and Prevention, Beijing, China.

^2^Collaborative Innovation Center for Diagnosis and Treatment of Infectious Diseases, Hangzhou, China.

^3^Center for Human Pathogen Collection, Chinese Center for Disease Control and Prevention, Beijing, China.

^4^Ma’anshan Center for Disease Control and Prevention, Ma’anshan, China.

^5^Laizhou Center for Disease Control and Prevention, Laizhou, China.

Running title: SXT/R391 in *Shewanella.*

Keywords: distribution, genetic characteristics, SXT/R391, integrative conjugative elements, *Shewanella*, China

Correspondence: Duochun Wang, wangduochun@icdc.cn

**Supplementary figure**

**
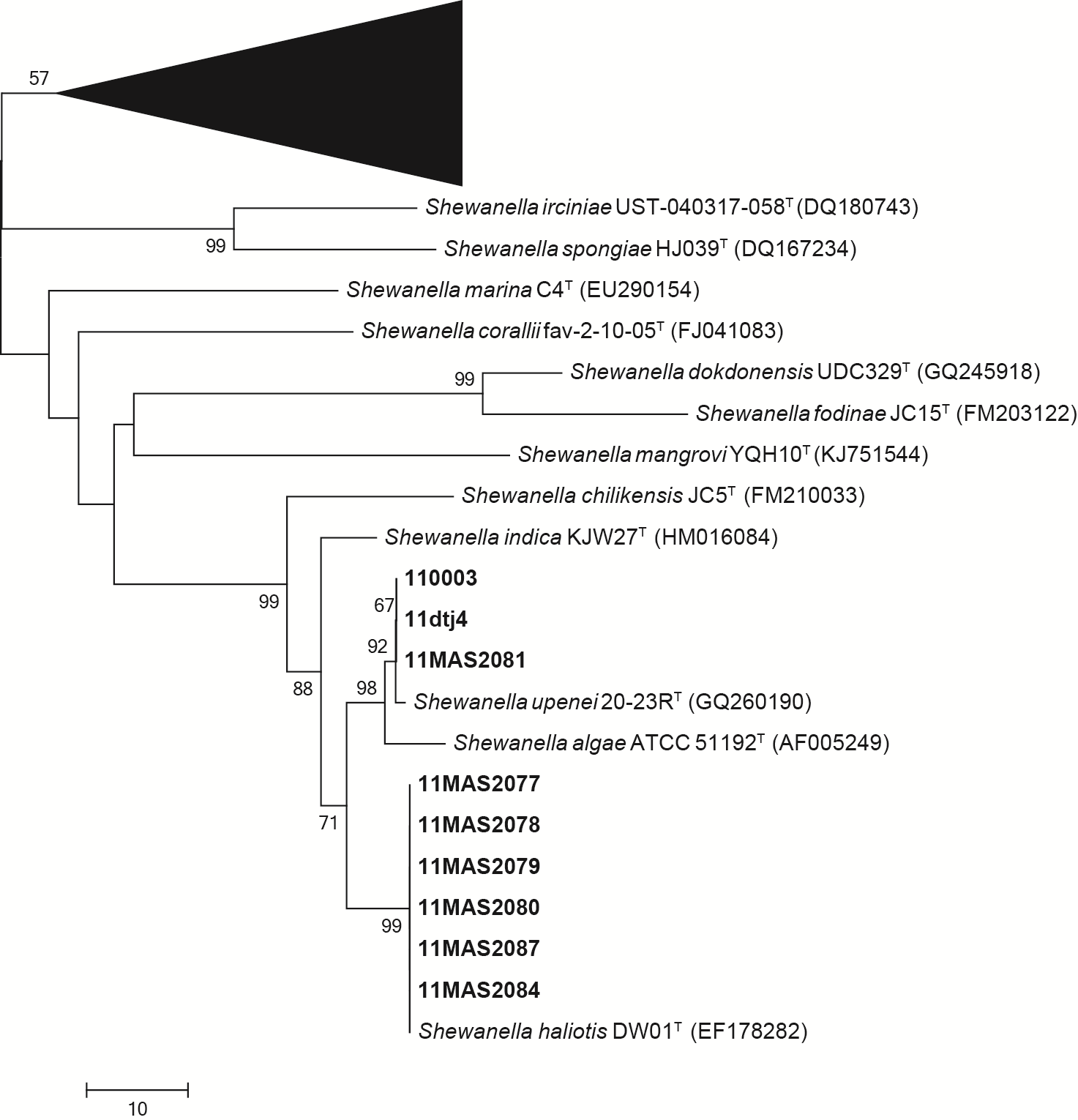
**

**Figure S1.** Phylogenetic tree constructed by the neighbour-joining method based on the 16S rRNA gene. The nucleotide sequences in the length of 1427 bp were aligned and all type strains of *Shewanella* species were included. Numbers at branch nodes indicated bootstrap values which was estimated by 1000 replicates and values>50% were shown. Bar, 10 indicated nucleotide substitutions per site. GenBank accession numbers of 16S rRNA gene sequences were in brackets. Bold font indicated nine *Shewanella* isolates collected from Dangtu county. The black triangle consisted of fifty-five *Shewanella* species which species names and GenBank accession numbers of 16S rRNA gene sequences were listed in supplementary table S2.

| **Table S1.** Twenty-four representative ICEs derived from different sequences scores and distinct evolutionary origins for phylogenetic analysis of core genes. | | | | | |
| --- | --- | --- | --- | --- | --- |
| Strain name | ICEs name | Isolation place | Isolation source | Isolation year | Accession number |
| *Alteromonas macleodii* MED64 | ICE^MED64^ | Aegean Sea of Lebanon | Water | 2000 | CP004848.1 |
| *Alteromonas macleodii* UM7 | ICE^UM7^ | Ionian Sea, Urania Basin | Water | 1998 | CP004853.1 |
| *Photobacterium damselae* subsp. *piscicida* PC554.2 | ICE*Pda*Spa1 | Galicia, Spain | Fish | 2001 | AJ870986.2 |
| *Proteus mirabilis* 09MAS2410 | ICE*Pmi*CHN2410 | Anhui, China | Stool | 2009 | KX243406.1 |
| *Proteus mirabilis* HI4320 | ICE*Pmi*Usa1 | Maryland, USA | Patient | 1986 | AM942759.1 |
| *Proteus mirabilis* PM13C04 | ICE*Pmi*Chn1 | Hubei, China | Chicken | 2013 | KT962845.1 |
| *Proteus mirabilis* TJ1809 | ICE*Pmi*CHN1809 | Tianjin, China | Stool | 2013 | KX243413.1 |
| *Proteus mirabilis* TJ3300 | ICE*Pmi*CHN3300 | Tianjin, China | Stool | 2013 | KX243415.1 |
| *Providencia alcalifaciens* Ban1 | ICE*Pal*Ban1 | Bangladesh | - | 1999 | GQ463139.1 |
| *Providencia rettgeri* R391 | R391 | Pretoria, South Africa | Stool | 1967 | AY090559.1 |
| *Providencia stuartii* ATCC 33672 | ICE^ATCC 33672^ | - | - | - | CP008920.1 |
| *Shewanella putrefaciens* W3-18-1 | ICE*Spu*PO1 | Paciﬁc Ocean | Marine sediment | 2000 | CP000503.1 |
| *Vibrio alginolyticus* A056 | ICE*Val*A056-1 | Guangdong, China | Shrimp | 2003 | KR231688.1 |
| *Vibrio alginolyticus* HN492 | ICE*Val*HN492 | Hainan, China | Seawater | 2008 | KT072769.1 |
| *Vibrio cholerae* 1-010118-075 | ICE*Vch*Mex1 | San Luis Potosi, Mexico | Sewage | 2001 | GQ463143.1 |
| *Vibrio cholerae* Ban5 | ICE*Vch*Ban5 | Bangladesh | Clinical sample | 1998 | GQ463140.1 |
| *Vibrio cholerae* HC-1A2 | ICE*Vch*Hai2 | Haiti | Stool | 2010 | AJRO01000008.1 |
| *Vibrio cholerae* ICDC-2255 | ICE*Vch*CHN2255 | Hainan, China | Patient | 2008 | KT151660.1 |
| *Vibrio cholerae* ICDC-4210 | ICE*Vch*CHN4210 | Jiangxi, China | Patient | 1999 | KT151662.1 |
| *Vibrio cholerae* MJ-1236 | ICE*Vch*Ban9 | Matlab, Bangladesh | Patient | 1994 | CP001485.1 |
| *Vibrio cholerae* MO10 | SXT^MO10^ | Chennai, India | Clinical sample | 2002 | AY055428.1 |
| *Vibrio fluvialis* H-08942 | ICE*Vfl*Ind1 | Kolkata, India | Patient | 2002 | KM213605.1 |
| *Vibrio parahaemolyticus* CHN25 | ICE*Vpa*Chn1 | Shanghai, China | Shrimp | 2011 | CP010883.1 |
| *Vibrio parahaemolyticus* UCM-V493 | ICE^UCM-V493^ | Spain | Sediment | 2002 | CP007004.1 |

**Table S2.** *Shewanella* species information of 16S rRNA gene sequences of the black triangle in Figure S1.

| *Shewanella* species | Accession number | |
| --- | --- | --- |
| *Shewanella abyssi* c941^T^ | AB201475 |  |
| *Shewanella aestuarii* SC18^T^ | JF751044 |  |
| *Shewanella algicola* ST-6^T^ | FJ903681 |  |
| *Shewanella algidipiscicola* S13^T^ | AB205570 |  |
| *Shewanella amazonensis* SB2B^T^ | AF005248 |  |
| *Shewanella aquimarina* SW-120^T^ | AY485225 |  |
| *Shewanella arctica* IR12^T^ | GU564402 |  |
| *Shewanella atlantica* HAW-EB5^T^ | AY579752 |  |
| *Shewanella baltica* NCTC 10735^T^ | AJ000214 |  |
| *Shewanella basaltis* J83^T^ | EU143361 |  |
| *Shewanella benthica* ATCC 43992^T^ | X82131 |  |
| *Shewanella canadensis* HAW-EB2^T^ | AY579749 |  |
| *Shewanella colwelliana* ATCC 39565^T^ | AY653177 |  |
| *Shewanella decolorationis* S12^T^ | AJ609571 |  |
| *Shewanella denitrificans* OS217^T^ | AJ311964 |  |
| *Shewanella donghaensis* LT17^T^ | AY326275 |  |
| *Shewanella electrodiphila* MAR441^T^ | FR744784 |  |
| *Shewanella fidelis* KMM 3582^T^ | AF420312 |  |
| *Shewanella frigidimarina* ACAM 591^T^ | U85903 |  |
| *Shewanella gaetbuli* TF-27^T^ | AY190533 |  |
| *Shewanella gelidii* RZB5-4^T^ | KR080702 |  |
| *Shewanella gelidimarina* ACAM 456^T^ | U85907 |  |
| *Shewanella glacialipiscicola* T147^T^ | AB205571 |  |
| *Shewanella hafniensis* P010^T^ | AB205566 |  |
| *Shewanella halifaxensis* HAW-EB4^T^ | AY579751 |  |
| *Shewanella hanedai* ATCC 33224^T^ | U91590 |  |
| *Shewanella inventionis* KX27^T^ | KT781407 |  |
| *Shewanella japonica* KMM 3299^T^ | AF145921 |  |
| *Shewanella kaireitica* c931^T^ | AB094598 |  |
| *Shewanella litorisediminis* SMK1-12^T^ | JQ824139 |  |
| *Shewanella livingstonensis* NF22^T^ | AJ300834 |  |
| *Shewanella loihica* PV-4^T^ | DQ286387 |  |
| *Shewanella marinintestina* IK-1^T^ | AB081757 |  |
| *Shewanella marisflavi* SW-117^T^ | AY485224 |  |
| *Shewanella morhuae* U1417^T^ | AB205576 |  |
| *Shewanella olleyana* ACEM 9^T^ | AF295592 |  |
| *Shewanella oneidensis* MR-1^T^ | AF005251 |  |
| *Shewanella pacifica* KMM 3597^T^ | AF500075 |  |
| *Shewanella pealeana* ANG-SQ1^T^ | AF011335 |  |
| *Shewanella piezotolerans* WP3^T^ | AJ551090 |  |
| *Shewanella pneumatophori* SCRC-2738^T^ | AB204519 |  |
| *Shewanella profunda* LT13a^T^ | AY445591 |  |
| *Shewanella psychrophila* WP2^T^ | AJ551089 |  |
| *Shewanella putrefaciens* ATCC 8071^T^ | X82133 |  |
| *Shewanella sairae* SM2-1^T^ | AB081762 |  |
| *Shewanella schlegeliana* HRKA1^T^ | AB081760 |  |
| *Shewanella sediminis* HAW-EB3^T^ | AY579750 |  |
| *Shewanella seohaensis* S7-3^T^ | GU944672 |  |
| *Shewanella surugensis* c959^T^ | AB094597 |  |
| *Shewanella vesiculosa* M7^T^ | AM980877 |  |
| *Shewanella violacea* DSS12^T^ | D21225 |  |
| *Shewanella waksmanii* KMM 3823^T^ | AY170366 |  |
| *Shewanella woodyi* MS32^T^ | U91590 |  |
| *Shewanella xiamenensis* S4^T^ | FJ589031 |  |

| **Table S3.** The predicted ORFs of ICE*Sup*CHN110003 annotated by RAST. | | | | | | |
| --- | --- | --- | --- | --- | --- | --- |
| Locus | Start | Stop | Strand | Gene | Function | Region |
| ICE*Sup*CHN110003_001 | 481 | 669 | + |  | hypothetical protein |  |
| ICE*Sup*CHN110003_002 | 815 | 2833 | + | *mutL* | DNA mismatch repair protein MutL | VRII |
| ICE*Sup*CHN110003_003 | 4204 | 2963 | - | *int* | integrase |  |
| ICE*Sup*CHN110003_004 | 4475 | 4206 | - |  | hypothetical protein |  |
| ICE*Sup*CHN110003_005 | 5452 | 4478 | - |  | rod shape determination protein |  |
| ICE*Sup*CHN110003_006 | 5548 | 5715 | + |  | hypothetical protein |  |
| ICE*Sup*CHN110003_007 | 5760 | 5894 | + |  | hypothetical protein |  |
| ICE*Sup*CHN110003_008 | 5917 | 6360 | + |  | hypothetical protein |  |
| ICE*Sup*CHN110003_009 | 7543 | 6371 | - | *rumB* | error-prone, lesion bypass DNA polymerase V (UmuC) |  |
| ICE*Sup*CHN110003_010 | 7827 | 8420 | + | *tnp* | transposase | VRIII |
| ICE*Sup*CHN110003_011 | 8534 | 11512 | + | *tnpA* | mobile element protein |
| ICE*Sup*CHN110003_012 | 11627 | 11818 | + |  | hypothetical protein |
| ICE*Sup*CHN110003_013 | 11930 | 13423 | + | *tnpB* | mobile element protein |
| ICE*Sup*CHN110003_014 | 13517 | 14338 | + |  | type IV secretory pathway, VirD2 components (relaxase) |
| ICE*Sup*CHN110003_015 | 14555 | 15769 | + | *floR* | bicyclomycin resistance protein |
| ICE*Sup*CHN110003_016 | 15797 | 16102 | + |  | LysR family transcriptional regulator STM3121 |
| ICE*Sup*CHN110003_017 | 16214 | 16753 | + | *tnpB* | mobile element protein |
| ICE*Sup*CHN110003_018 | 17555 | 16725 | - | *strB* | aminoglycoside 3'-phosphotransferase 2 |
| ICE*Sup*CHN110003_019 | 18364 | 17561 | - | *strA* | aminoglycoside 3'-phosphotransferase |
| ICE*Sup*CHN110003_020 | 19240 | 18425 | - | *sul2* | dihydropteroate synthase |
| ICE*Sup*CHN110003_021 | 19711 | 21063 | + | *tnpA* | mobile element protein |
| ICE*Sup*CHN110003_022 | 22266 | 21055 | - | *tnp* | mobile element protein |
| ICE*Sup*CHN110003_023 | 23249 | 22980 | - | *rumB* | error-prone, lesion bypass DNA polymerase V (UmuC) |  |
| ICE*Sup*CHN110003_024 | 23706 | 23257 | - | *rumA* | error-prone repair protein UmuD |  |
| ICE*Sup*CHN110003_025 | 24348 | 25253 | + |  | DNA polymerase III, epsilon subunit-like protein |  |
| ICE*Sup*CHN110003_026 | 25467 | 25766 | + |  | hypothetical protein |  |
| ICE*Sup*CHN110003_027 | 26084 | 27019 | + |  | hypothetical protein |  |
| ICE*Sup*CHN110003_028 | 27057 | 30389 | + |  | DEAD/DEAH box helicase-like protein | HS5 |
| ICE*Sup*CHN110003_029 | 30393 | 31079 | + |  | methyl-accepting chemotaxis protein |
| ICE*Sup*CHN110003_030 | 31162 | 33108 | + |  | type III restriction-modification system methylation subunit |
| ICE*Sup*CHN110003_031 | 33118 | 36195 | + |  | type III restriction enzyme, res subunit |
| ICE*Sup*CHN110003_032 | 36269 | 37123 | + |  | mrr restriction system protein |
| ICE*Sup*CHN110003_033 | 37218 | 39368 | + | *traI* | conjugative transfer protein TraI, relaxase |  |
| ICE*Sup*CHN110003_034 | 39417 | 41237 | + | *traD* | IncF plasmid conjugative transfer protein TraD |  |
| ICE*Sup*CHN110003_035 | 41247 | 41807 | + |  | conjugative transfer protein 234 |  |
| ICE*Sup*CHN110003_036 | 41794 | 42429 | + |  | conjugative transfer protein s043 |  |
| ICE*Sup*CHN110003_037 | 43043 | 42456 | - |  | hypothetical protein | HS1 |
| ICE*Sup*CHN110003_038 | 43332 | 43613 | + | *traL* | IncF plasmid conjugative transfer pilus assembly protein TraL |  |
| ICE*Sup*CHN110003_039 | 43610 | 44236 | + | *traE* | IncF plasmid conjugative transfer pilus assembly protein TraE |  |
| ICE*Sup*CHN110003_040 | 44220 | 45116 | + | *traK* | IncF plasmid conjugative transfer pilus assembly protein TraK |  |
| ICE*Sup*CHN110003_041 | 45119 | 46408 | + | *traB* | IncF plasmid conjugative transfer pilus assembly protein TraB |  |
| ICE*Sup*CHN110003_042 | 46405 | 47055 | + | *traV* | conjugative transfer protein TraV |  |
| ICE*Sup*CHN110003_043 | 47052 | 47426 | + | *traA* | conjugative transfer protein TraA |  |
| ICE*Sup*CHN110003_044 | 47502 | 48230 | + |  | hypothetical protein | HS2 |
| ICE*Sup*CHN110003_045 | 48418 | 49110 | + |  | thiol:disulfide involved in conjugative transfer |  |
| ICE*Sup*CHN110003_046 | 49110 | 51509 | + | *traC* | IncF plasmid conjugative transfer pilus assembly protein TraC |  |
| ICE*Sup*CHN110003_047 | 51502 | 51849 | + |  | conjugative transfer protein 345 |  |
| ICE*Sup*CHN110003_048 | 51833 | 52345 | + | *trhF* | conjugative signal peptidase TrhF |  |
| ICE*Sup*CHN110003_049 | 52356 | 53480 | + | *traW* | IncF plasmid conjugative transfer pilus assembly protein TraW |  |
| ICE*Sup*CHN110003_050 | 53512 | 54492 | + | *traU* | IncF plasmid conjugative transfer pilus assembly protein TraU |  |
| ICE*Sup*CHN110003_051 | 54495 | 58187 | + | *traN* | IncF plasmid conjugative transfer protein TraN |  |
| ICE*Sup*CHN110003_052 | 58610 | 60310 | + |  | predicted ATP-dependent endonuclease of the OLD family | HS4 |
| ICE*Sup*CHN110003_053 | 60312 | 61946 | + |  | ATP-dependent DNA helicase UvrD/PcrA |
| ICE*Sup*CHN110003_054 | 62824 | 62141 | - |  | endonuclease I precursor |
| ICE*Sup*CHN110003_055 | 63551 | 62949 | - |  | hypothetical protein |  |
| ICE*Sup*CHN110003_056 | 63727 | 63584 | - |  | hypothetical protein |  |
| ICE*Sup*CHN110003_057 | 63919 | 64245 | + |  | hypothetical protein |  |
| ICE*Sup*CHN110003_058 | 64261 | 64680 | + | *ssb* | single-stranded DNA-binding protein |  |
| ICE*Sup*CHN110003_059 | 64760 | 65578 | + | *bet* | recombination protein BET |  |
| ICE*Sup*CHN110003_060 | 65661 | 65804 | + |  | hypothetical protein |  |
| ICE*Sup*CHN110003_061 | 65865 | 66881 | + |  | hypothetical protein |  |
| ICE*Sup*CHN110003_062 | 67091 | 68050 | + |  | aerobic cobaltochelatase CobS subunit |  |
| ICE*Sup*CHN110003_063 | 68050 | 68817 | + |  | hypothetical protein |  |
| ICE*Sup*CHN110003_064 | 68916 | 69869 | + |  | cobalamine biosynthesis protein |  |
| ICE*Sup*CHN110003_065 | 69931 | 70371 | + |  | hypothetical protein |  |
| ICE*Sup*CHN110003_066 | 70441 | 72096 | + |  | plasmid associated gene product APECO1_O1R37 |  |
| ICE*Sup*CHN110003_067 | 72181 | 72678 | + | *radC* | DNA repair protein RadC |  |
| ICE*Sup*CHN110003_068 | 72678 | 73019 | + |  | hypothetical protein |  |
| ICE*Sup*CHN110003_069 | 73110 | 74183 | + |  | putative primase |  |
| ICE*Sup*CHN110003_070 | 74271 | 74978 | + |  | hypothetical protein |  |
| ICE*Sup*CHN110003_071 | 75176 | 80842 | + |  | serine protease-like protein | HS3 |
| ICE*Sup*CHN110003_072 | 80970 | 81914 | + | *traF* | IncF plasmid conjugative transfer pilus assembly protein TraF |  |
| ICE*Sup*CHN110003_073 | 81917 | 83305 | + | *traH* | IncF plasmid conjugative transfer pilus assembly protein TraH |  |
| ICE*Sup*CHN110003_074 | 83309 | 86878 | + | *traG* | IncF plasmid conjugative transfer protein TraG |  |
| ICE*Sup*CHN110003_075 | 86899 | 87117 | + |  | hypothetical protein |  |
| ICE*Sup*CHN110003_076 | 87930 | 87397 | - | *setC* | transcriptional activator |  |
| ICE*Sup*CHN110003_077 | 88226 | 87927 | - | *setD* | transcriptional activator |  |
| ICE*Sup*CHN110003_078 | 88771 | 88223 | - |  | soluble lytic murein transglycosylase and related regulatory proteins | |
| ICE*Sup*CHN110003_079 | 89420 | 88758 | - |  | hypothetical protein |  |
| ICE*Sup*CHN110003_080 | 90276 | 89407 | - |  | hypothetical protein |  |
| ICE*Sup*CHN110003_081 | 90583 | 90332 | - |  | hypothetical protein |  |
| ICE*Sup*CHN110003_082 | 90701 | 91348 | + | *setR* | putative cI prophage repressor protein |  |
